# Nuclear Receptor Expression and Function in Human Lung Cancer Pathogenesis

**DOI:** 10.1371/journal.pone.0134842

**Published:** 2015-08-05

**Authors:** Jihye Kim, Mitsuo Sato, Jong-Whan Choi, Hyun-Won Kim, Byung-Il Yeh, Jill E. Larsen, John D. Minna, Jeong-Heon Cha, Yangsik Jeong

**Affiliations:** 1 Department of Biochemistry, Wonju College of Medicine, Yonsei University, Wonju, Gangwon-do, Republic of Korea; 2 Institute of Lifestyle Medicine, Wonju College of Medicine, Yonsei University, Wonju, Gangwon-do, Republic of Korea; 3 Nuclear Receptor Research Consortium, Wonju College of Medicine, Yonsei University, Wonju, Gangwon-do, Republic of Korea; 4 Department of Oral Biology, Oral Cancer Research Institute, Oral Science Research Center, BK21 Project, Research Center for Orofacial Hard Tissue Regeneration, Yonsei University College of Dentistry, Seoul, Republic of Korea; 5 Department of Pharmacology, University of Texas Southwestern Medical Center, Dallas, Texas, United States of America; 6 Department of Internal Medicine, University of Texas Southwestern Medical Center, Dallas, Texas, United States of America; 7 Hamon Center for Therapeutic Oncology Research, University of Texas Southwestern Medical Center, Dallas, Texas, United States of America; 8 The Simmons Comprehensive Cancer Center, University of Texas Southwestern Medical Center, Dallas, Texas, United States of America; 9 Department of Respiratory Medicine, Nagoya University Graduate School of Medicine, Showa-ku, Nagoya, Japan; Kyung Hee University, KOREA, REPUBLIC OF

## Abstract

Lung cancer is caused by combinations of diverse genetic mutations. Here, to understand the relevance of nuclear receptors (NRs) in the oncogene-associated lung cancer pathogenesis, we investigated the expression profile of the entire 48 NR members by using QPCR analysis in a panel of human bronchial epithelial cells (HBECs) that included precancerous and tumorigenic HBECs harboring oncogenic *K-ras^V12^* and/or *p53* alterations. The analysis of the profile revealed that oncogenic alterations accompanied transcriptional changes in the expression of 19 NRs in precancerous HBECs and 15 NRs according to the malignant progression of HBECs. Amongst these, peroxisome proliferator-activated receptor gamma (PPARγ), a NR chosen as a proof-of-principle study, showed increased expression in precancerous HBECs, which was surprisingly reversed when these HBECs acquired full *in vivo* tumorigenicity. Notably, PPARγ activation by thiazolidinedione (TZD) treatment reversed the increased expression of pro-inflammatory cyclooxygenase 2 (COX2) in precancerous HBECs. In fully tumorigenic HBECs with inducible expression of PPARγ, TZD treatments inhibited tumor cell growth, clonogenecity, and cell migration in a PPARγ-sumoylation dependent manner. Mechanistically, the sumoylation of liganded-PPARγ decreased COX2 expression and increased 15-hydroxyprostaglandin dehydrogenase expression. This suggests that ligand-mediated sumoylation of PPARγ plays an important role in lung cancer pathogenesis by modulating prostaglandin metabolism.

## Introduction

The nuclear receptor superfamily comprises 48 members of transcription factors that are mostly ligand-activated and play crucial roles in diverse physiological processes including metabolism, development, and differentiation in the body. Dysregulation of NR pathways causes severe chronic diseases such as diabetes [1–3], atherosclerosis [4, 5], and several types of cancer [6–8]. The expression of the entire NR superfamily has been investigated in various physiological and pathological conditions including cellular differentiation models [9–12], mouse anatomical systems [1], NCI60 panel of human cancer cell lines [6], and human lung cancer cell lines and patient samples [7, 8]. These studies have revealed that subsets of NRs are not only associated with specified tissue physiology, but are also relevant to functional differentiation into specific cellular lineages [13, 14]. Furthermore, the genetic signature of the NR superfamily or of individual NR members is a prognostic biomarker for lung cancer, and some NRs are druggable targets that may be pharmacologically developed into potential cancer treatments [6–8, 15–19]. Indeed, several lines of evidence show that individual NRs are associated with the onset and development as well as the treatment or chemoprevention of cancer. For instance, overexpression of retinoic acid receptor alpha (RARα) due to its fusion to PML (RARα/PML) and estrogen receptor alpha (ERα) expression cause onset of leukemia and breast cancer progression, respectively [20–22]. Targeting ERα using the selective estrogen receptor modulator (SERM) tamoxifen or raloxifene and blockade or ablation of dihydrotestosterone (DHT), which is the strongest endogenous ligand for androgen receptor (AR), are well-known therapeutic schemes in the cancer clinics to treat the corresponding cancers [19, 23–25]. While SERMs have been widely used for treatment of breast cancer or even clinically evaluated as chemopreventive agents against the incidence of breast cancer, a high risk of uterine and endometrium cancer incidence has been reported previously [26, 27]. Similarly, although the anti-diabetic drug TZDs are in clinical trials for lung cancer treatment, molecular function of the TZD-activated PPARγ has not been clearly defined yet as an anti-tumorigenic factor in lung cancer, or even argued as a tumor-promoting factor in other types of cancers, i.e. breast cancer and prostate cancer [28–30].

We have developed a preclinical model involving immortalized normal human bronchial epithelial cells (HBECs) that can be genetically manipulated with specific oncogenic changes to study lung cancer pathogenesis and the development of new targeted approaches for the early diagnosis, prevention, and treatment of lung cancer. Recently, we have been able to develop fully progressed and tumorigenic models with HBECs based on manipulation of p53, KRAS, and c-myc [31]. These precancerous and fully tumorigenic models provide an ideal system to study the role of NRs in lung cancer pathogenesis including preneoplasia and tumor formation.

In this study, we profiled the mRNA expression of all 48 NRs in this HBEC oncogene-induced lung cancer pathogenesis model [32, 33]. This allowed us to identify a key role of PPARγ in lung cancer pathogenesis. In a mechanistic proof-of-principle study, we found that PPARγ sumoylation is important for the anti-tumorigenic effect of TZDs in lung cancer pathogenesis. This study provides insight into a biochemical modification of PPARγ, which is useful for the understanding of lung cancer pathogenesis and also indicates the power of this preclinical system to study the role of NRs in lung cancer pathogenesis and these results should be of clinical translational value.

## Materials and Methods

### Cell culture

As previously reported, normal human bronchial epithelial cells were immortalized by CDK4 and hTERT (HBEC-KT), followed by further stable introduction of oncogenic alterations including K-ras^v12^ overexpression, knockdown of p53, or both [31–33]. A series of HBEC-KTs included HBEC-KT, HBEC-KTZ, HBEC-KT+pSRZ+pLenti-*K-ras* ^*v12*^ (HBEC-KTR_L_), HBEC KT+pSRZ-*p53* shRNA +pLenti-*LacZ* (HBEC-KT53), and HBEC KT+pSRZ-*p53* shRNA +pLenti-*K-ras* ^*v12*^ (HBEC-KTR_L_53), where pSRZ and pLenti represent stable shRNA and lentiviral vectors, respectively [31]. Immortalized HBECs and tumorigenic HBEC clones (C1 and C5) were cultured in K-SFM (Gibco, Gaithersburg, MD, USA) supplemented with 50 μg/ml of Bovine Pituitary Extract without epidermal growth factor (EGF) and RPMI supplemented with 10% fetal bovine serum, respectively.

### Molecular cloning and stable cell line

Both PPARγ and enhanced green fluorescence protein (EGFP) genes flanked by internal ribosomal entry site were bicistronically constructed under the control of tetracycline-inducible (Tet/On) cytomegalovirus promoter of lentiviral vector (Invitrogen, Carlsbad, CA, USA). Site-directed mutations were introduced into both sumoylation sites (K79R and K367R for PPARγ1; K107R and K395R for PPARγ2) in PPARγ. Lentiviruses were produced and transduced into tumorigenic HBEC-C1 cell lines. Further screening process was performed to select a HBEC-C1-PPARγ clone in which both PPARγ and EGFP expression are tightly regulated upon tetracycline treatment.

### Immunoblot analysis

A panel of immortalized HBEC cell lines was cultured in the presence or absence of PPARγ agonist troglitazone or pioglitazone for 48 hrs and then total cell lysates were prepared as described previously [7]. Primary antibodies for cell signaling included for antibodies against p53 (sc-126, Santa Cruz Biotechnology, Santa Cruz, CA, USA), K-ras (sc-30, Santa Cruz), phospho-MEK1/2 (#9121, Cell Signaling Technology, Beverly, MA, USA), MEK1/2 (#9122, Cell Signaling), COX2 (sc-19999, Santa Cruz), phospho-ERK1/2 (#9101, Cell Signaling), ERK1/2 (#9102, Cell Signaling), β-actin (Ab6276, Abcam, Cambridge, Cambridgeshire, UK), lamin A/C (sc-7292, Santa Cruz), and PPARγ (#2435, Cell signaling). Primary antibodies for cell cycle progression included antibodies against cyclinD1 (#2926, Cell Signaling), cyclin A (sc-239, Santa Cruz), p16^INK^ (sc-468, Santa Cruz), and p21^WAF1^ (OP64, Calbiochem, La Jolla, CA, USA).

### Cell growth and colony formation assay

Cell counting assay was performed for measuring cell growth responses. For cell counting assay, two hundreds of HBECs were seeded into 96-well plates in a final media volume of 100 μl per well, followed by troglitazone treatment at concentrations of 3 μM in the presence of 100 nM synthetic RXR ligand LG268. Cell number was counted at five days post-treatment. Relative % growth was normalized for each dose by vehicle treatment. For colony formation assay, five thousands of HBEC cells were splitted into 6-well plates and treated with PPARγ or PPARα ligands. Colonies were stained with methylene blue after 7 to 10 days ligand treatment.

### Cell migration assay

HBECs with inducible expression of wt-PPARγ or SUMO-PPARγ were seeded in 6-well plates and fully grown with 100% confluency, followed by mitomycin C treatment to suppress cell proliferation. A wound was formed by scratching cell layer with sterile pipette tip. The cells were then incubated in RPMI 1640 media containing 5% fetal bovine serum with or without tetracyclin induction for the receptor expression, and followed by ligand treatment. Cell migration was measured after 24hrs of PPARγ ligand treatment by counting number of cells migrated in the wounded areas.

### Luciferase report assay

HEK 293 cells were co-transfected with the equivalent amount (15 ng) of either control plasmid or expression plasmids for wildtype (wt-PPARγ) or sumoylation mutant (SUMO-PPARγ) of PPARγ in combination with a luciferase reporter plasmid of PPAR response element (TK-PPRE3x-Luc, 50 ng) and a beta-galactosidase plasmid (β-Gal, 20 ng) for normalization of transfection efficiency. Cells were then treated with vehicle (EtOH), 1 μM of pioglitazone or troglitazone and assayed for luciferase activity.

### Reverse transcription and QPCR assay

All RNAs were prepared using the Qiagen kit and reverse-transcribed into cDNA for QPCR assay (TaqMan method) as previously described [12]. The reverse transcription of 2 μg total RNA in 100 μl reaction volume was further adjusted to a 200 μl final volumes. The SDS version 2.1 software was used to detect the real-time PCR reaction performed in the ABI 7900HT system. Efficiency-corrected standard curve assays were optimized for nonbiased, multiplate comparison as previously described [8].

### QPCR data analysis

A Macro was created to analyze the raw data imported into Microsoft Excel. The PCR efficiency (e), was calculated as e = 10^[-1/slope]^ where the slope is obtained from the standard curve by SDS 2.1 software, for the 18S reference and NR of interest. The macro included the following statistical calculations. The average (avg.) quantity for individual samples was derived from average relative quantity of triplicates, where the quantity is equal to e^-Ct^ (i.e., *quantity* = (10 ^[-1/slope]^)^-Ct^). The coefficient of variation (CV) can be further obtained by dividing standard deviation of the average (STDEV) by the average quantity (avg.), CV = stdev/avg. The statistical outlier point was excluded if it was > 17% CV. By using these quantities for both 18S and NR of interest, the normalized value for each NR expression was further calculated from division of NR quantity avg by reference quantity avg. (i.e., normalized value = NR quantity avg/ reference quantity avg). Furthermore, the standard deviation for the normalized value was calculated as S.D. = (normalized value) X {(CV of reference)^2^ + (CV of NR)^2^}^1/2^.

## Results

### Characterization of immortalized HBECs

**Fig 1:**
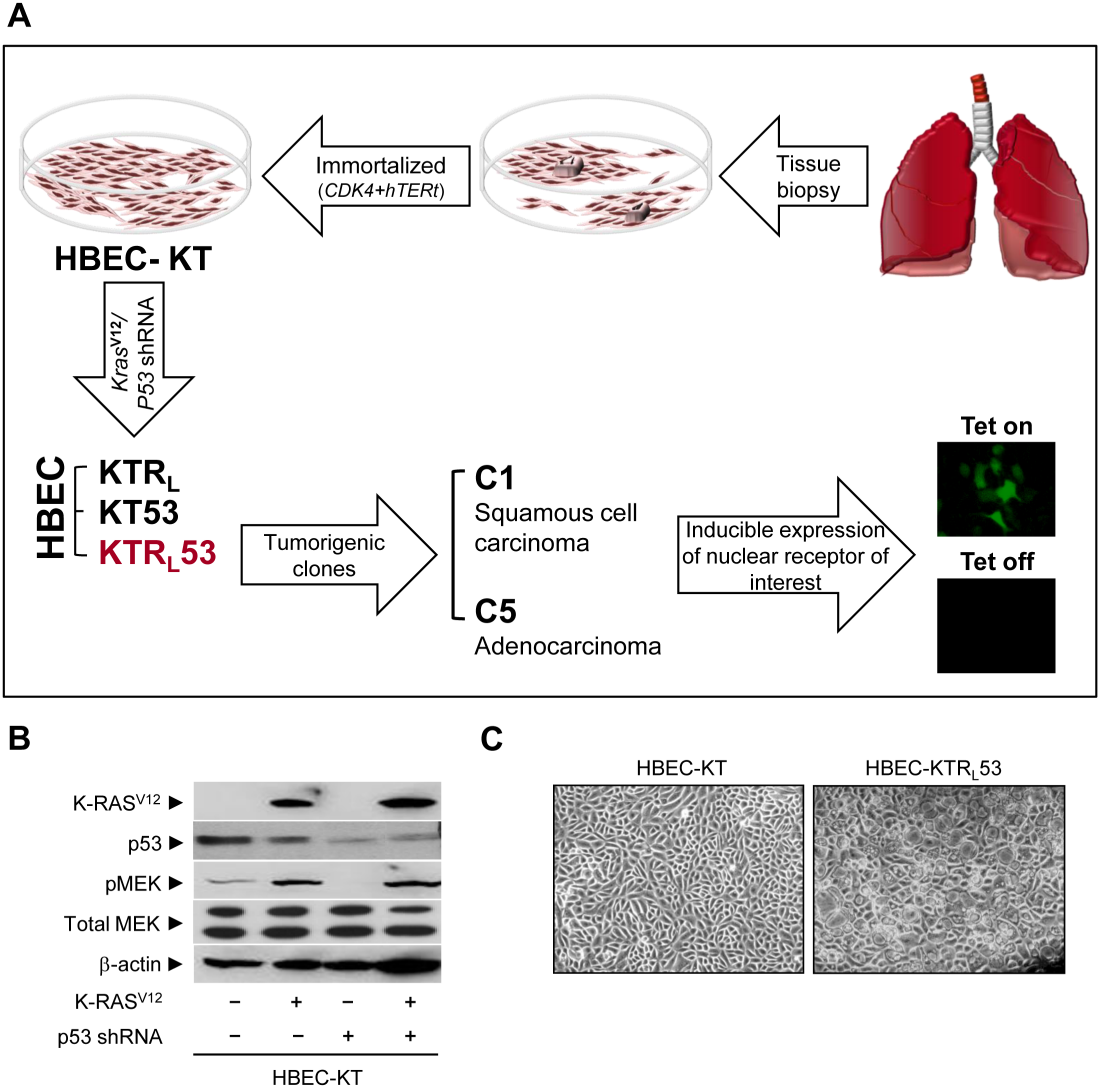
Characterization of human bronchial epithelial cells. (A) Schematics to generate a panel of HBEC cells. (B, C) *In vitro* characterization of HBECs. (B) Immunoblot assays were performed for the expression of K-ras^V12^, p53, pMEK, total MEK, and beta-actin in HBEC cells. (C) A microscopic view of HBEC cells (magnification, 1x). Note that HBEC-KT stands for HBEC cell lines immortalized by *CDK4* plus *hTERt*; KTR_L_, KT plus oncogenic *K-ras* ^*V12*^; KT53, KT plus *p53* knock-down; KTR_L_53, KTR_L_ plus *p53* knock-down.

The overall schema for generating immortalized and tumorigenic HBECs is shown in Fig 1A. To understand the effect of oncogenic alterations on the tumorigenic potential of bronchial epithelial cells, we previously generated a panel of immortalized HBECs harboring either *K-ras* ^*V12*^ expression, p53 knockdown, or both changes, which are major mutations in lung cancer [32, 33]. Using a mouse xenograft model, HBEC clones, C1 and C5, were identified to be tumorigenic and characterized. Stable knockdown of p53 was confirmed for both mRNA and protein expression using QPCR assay and immunoblot analysis, respectively (Fig 1B and S1 Fig). The activity of oncogenic *K-ras* ^*V12*^ stably introduced into the immortalized HBEC cell lines was confirmed by phosphorylation of MEK, a downstream target kinase of K-ras (Fig 1B). These genetic changes clearly induced a vacuole-like cellular morphological change that appeared to be cellular senescence, which is consistent with results from a previous report [31] (Fig 1C).

### NR expression in the HBEC panel

**Fig 2:**
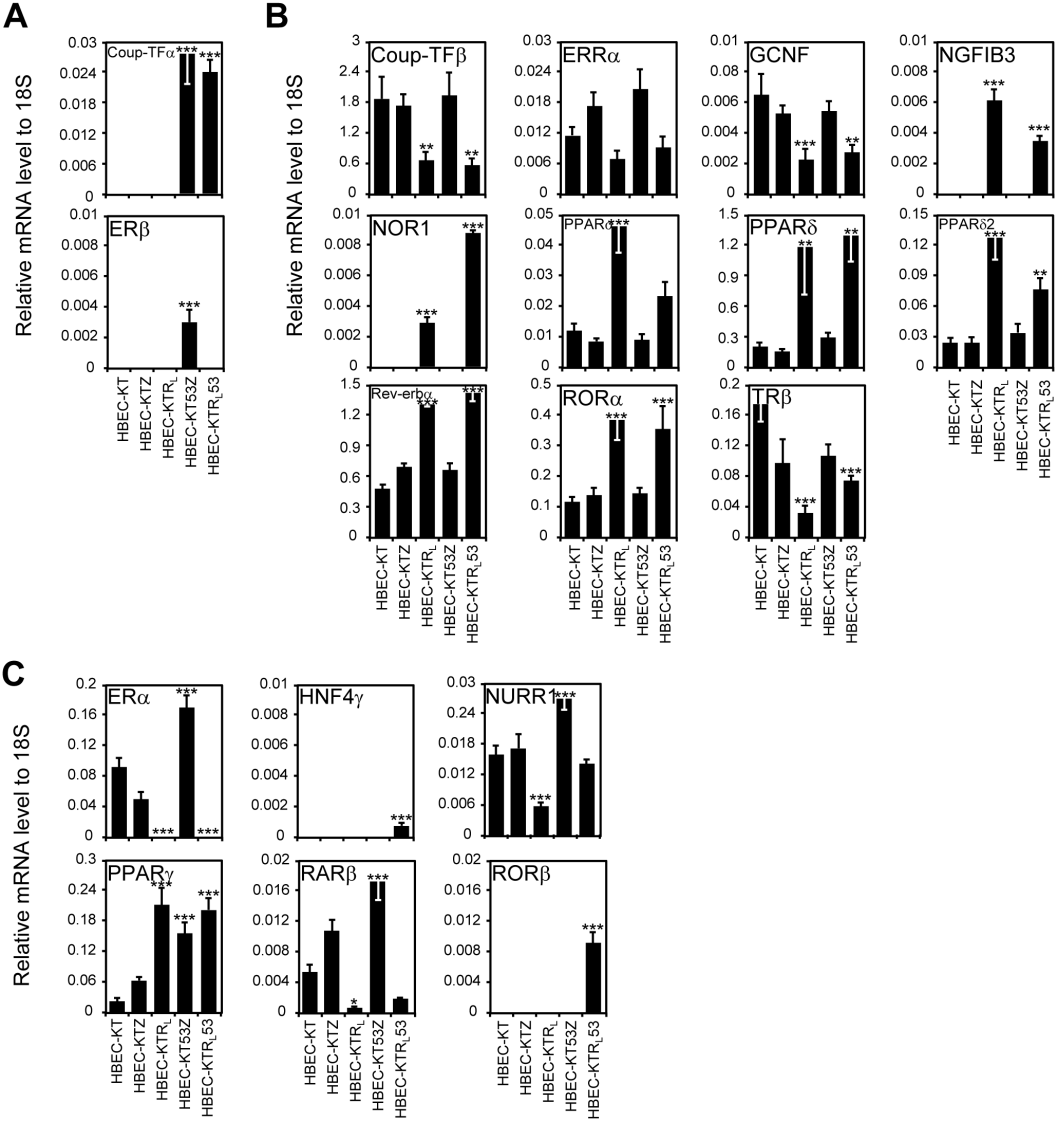
Expression profile of the NR superfamily in immortalized HBEC panel. The QPCR assay was performed for mRNA expression of the entire NR superfamily in immortalized HBEC panel. (A) *p53* knockdown-dependent expression. (B) Oncogenic *K-ras* ^*V12*^-dependent expression. (C) *p53* knockdown and *K-ras* ^*V12*^-dependent expression. Note that HBEC-KT stands for HBEC cell lines immortalized by *CDK4* plus *hTERT*; KTZ, KT plus control plasmid with zeocin selection marker; KTR_L_, KT plus oncogenic *K-ras* ^*V12*^; KT53Z, KTZ plus *p53* knock-down; KTR_L_53, KTR_L_ plus *p53* knockdown. Data represent the mean ± SD (n = 3). Asterisks show statistically significant points as evaluated by *ANOVA*. *P < 0.05, **P < 0.01 and ***P < 0.001 compared to HBEC-KT.

Since we recently demonstrated that the expression pattern for the 48 NRs is a prognostic biomarker set as well as potentially being therapeutic targets for lung cancer [6–8], we wondered if any NRs are associated with lung cancer pathogenesis. Therefore, to explore whether the introduction of *K-ras* ^*V12*^ and *p53* oncogenic changes affected the expression of NRs in human lung bronchial epithelial cells, we first profiled the mRNA expression of all 48 members of the NR superfamily by QPCR in the isogenic HBEC panel that is oncogenically well-defined and composed of genetically identical bronchial epithelial cell lines (Fig 2 and S2 Fig). We found 31 out of 50 NRs (including PPARδ2 and PPARγ2, isoforms of PPARδ and PPARγ, respectively) to exhibit no differences in the isogenic panel (either had no expression or no change in expression) (S2 Fig). By contrast, 19 NRs showed distinct expression patterns across the isogenic HBEC panels, which fell into three different groups. The first group (p53 dependent) included two members, chicken ovalbumin upstream promoter-transcription factor (Coup-TF)α, estrogen receptor(ER)β, NRs showing a p53-dependent expression pattern (Fig 2A). The second group was represented by NRs with a K-ras^V12^-dependent expression pattern including Coup-TFβ, estrogen-related receptor (ERR)α, germ cell nuclear factor (GCNF), nerve growth factor induced gene B (NGFIB)3, neuron-derived orphan receptor 1 (NOR1), PPARα, PPARδ, PPARδ2, reverse-erb (Rev-erb)α, and retinoic acid-related orphan receptor (ROR)α, thyroid hormone receptor (TR)β (Fig 2B). The third group (K-ras^V12^ and p53 dependent) were ERα, hepatocyte nuclear factor 4 (HNF4)γ, nur-related factor 1 (NURR1), PPARγ, Retinoid acid receptor (RAR)β, RAR-related orphan receptor (ROR)β, which were NRs with a dual oncogene-dependent expression pattern (Fig 2C). In line with the results from our previous report in which NR expression was highly associated with lung cancer progression [7], this result supports the notion that subsets of NRs could also be involved in lung cancer pathogenesis induced by *K-ras* ^*V12*^ overexpression and/or loss of p53 function.

### PPARγ activation reversed the oncogenic *K-ras* ^*V12*^-induced expression of COX2 protein

**Fig 3:**
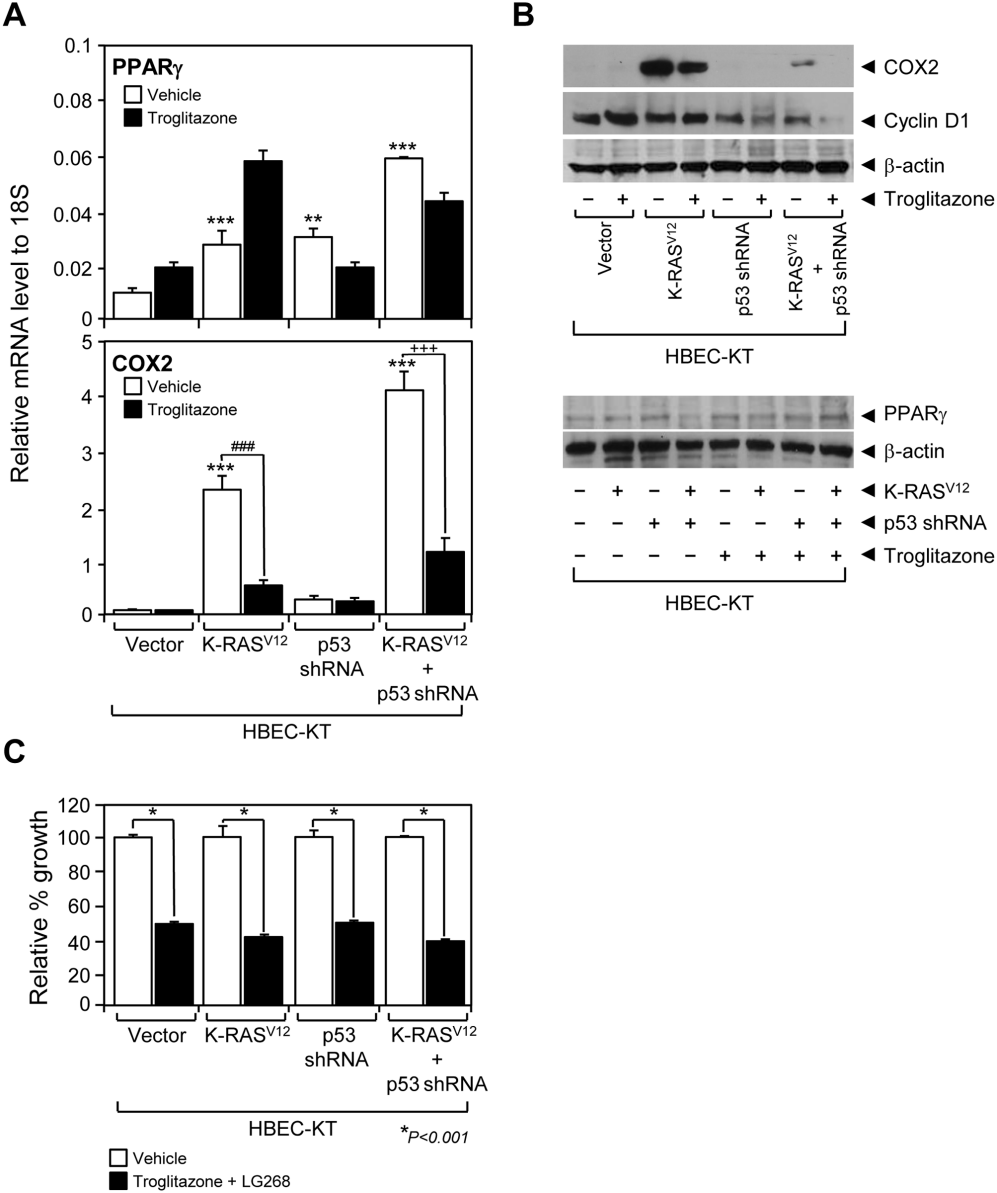
Functional evaluation of PPARγ in immortalized HBECs. (A) PPARγ and COX2 mRNA expression was measured using QPCR assay in HBEC lines with or without troglitazone treatment. (B) Immunoblots using antibodies against COX2, cyclin D1, PPARγ or beta-actin were used to measure the corresponding protein expression in the HBEC panel when treated or not treated with 1 μM of troglitazone. (C) Growth response of HBEC-KT cell lines to the combined treatment of PPARγ ligand troglitazone (3 μM) and RXR ligand LG268 (100 nM). Data represent the mean ± SD (n = 3). Asterisks show statistically significant points as evaluated by *ANOVA*. *P < 0.05, **P < 0.01 and ***P < 0.001 compared to HBEC-KT control, ^###^ *P* < 0.001 compared to K-ras^V12^ control, ^+++^ *P* < 0.001 compared to K-ras^V12^+p53shRNA control.

Since the PPARγ has been relatively well-studied amongst other NRs in diverse physiological functions (e.g., adipocyte differentiation, inflammation control) and its ligand TZDs available in the clinic of type II diabetes, we next decided to investigate the molecular study of PPARγ, as a proof-of-concept, out of the 48 NRs in the molecular pathogenesis of lung cancer [11, 34, 35]. Consistent with previous reports showing that COX2 expression is associated with lung cancer progression [36, 37], we observed a dramatic increase in COX2 expression in HBECs modified to contain oncogenic *K-ras* ^*V12*^ (Fig 3A and 3B). While PPARγ mRNA increased in parallel with COX2 expression, PPARγ protein expressions were comparable in all 4 HBEC cells suggesting posttranscriptional regulation of PPARγ mRNA (Fig 3B). Given that PPARγ plays a significant role in anti-inflammatory response [34, 35], we wanted to test if PPARγ activation inhibits the pro-inflammatory COX2 expression in the lung cancer pathogenesis model. Indeed, the PPARγ agonist troglitazone reversed the increased expression of COX2 mRNA and protein (Fig 3A and 3B). Together with the PPARγ inhibition of lung cancer proliferation as previously reported [38, 39], this result suggests that PPARγ suppression of COX2 expression might be important in modulating oncogene-induced malignant transformation of HBECs into lung cancer. With loss of p53 function, the expression of cyclin D1 protein, a key factor in cell cycle progression from G1 to S phase, decreased in HBECs with p53 knockdown and decreased even more in HBECs with dual *p53* and *K-ras* ^*V12*^ oncogenic alterations coupled with troglitazone treatment (Fig 3B). By contrast, cyclin D1 exhibited no change in HBECs with only K-ras^V12^ expression with or without troglitazone. These data suggest that during precancerous lesions (typified by the HBEC cells with *p53* loss and *K-ras* ^*V12*^ changes), PPARγ is expressed and its ligand-dependent activation can lead to dramatic changes in COX-2 and cyclin D1 expression (Fig 3A and 3B). However, troglitazone treatment showed equal inhibition of HBEC-KT proliferation across the isogenic panel (Fig 3C). This suggests that additional factors, along with COX-2 and cyclin D1, might be involved in PPARγ-mediated growth suppression of HBEC cells.

### Characterization of tumorigenic HBEC clones

**Fig 4:**
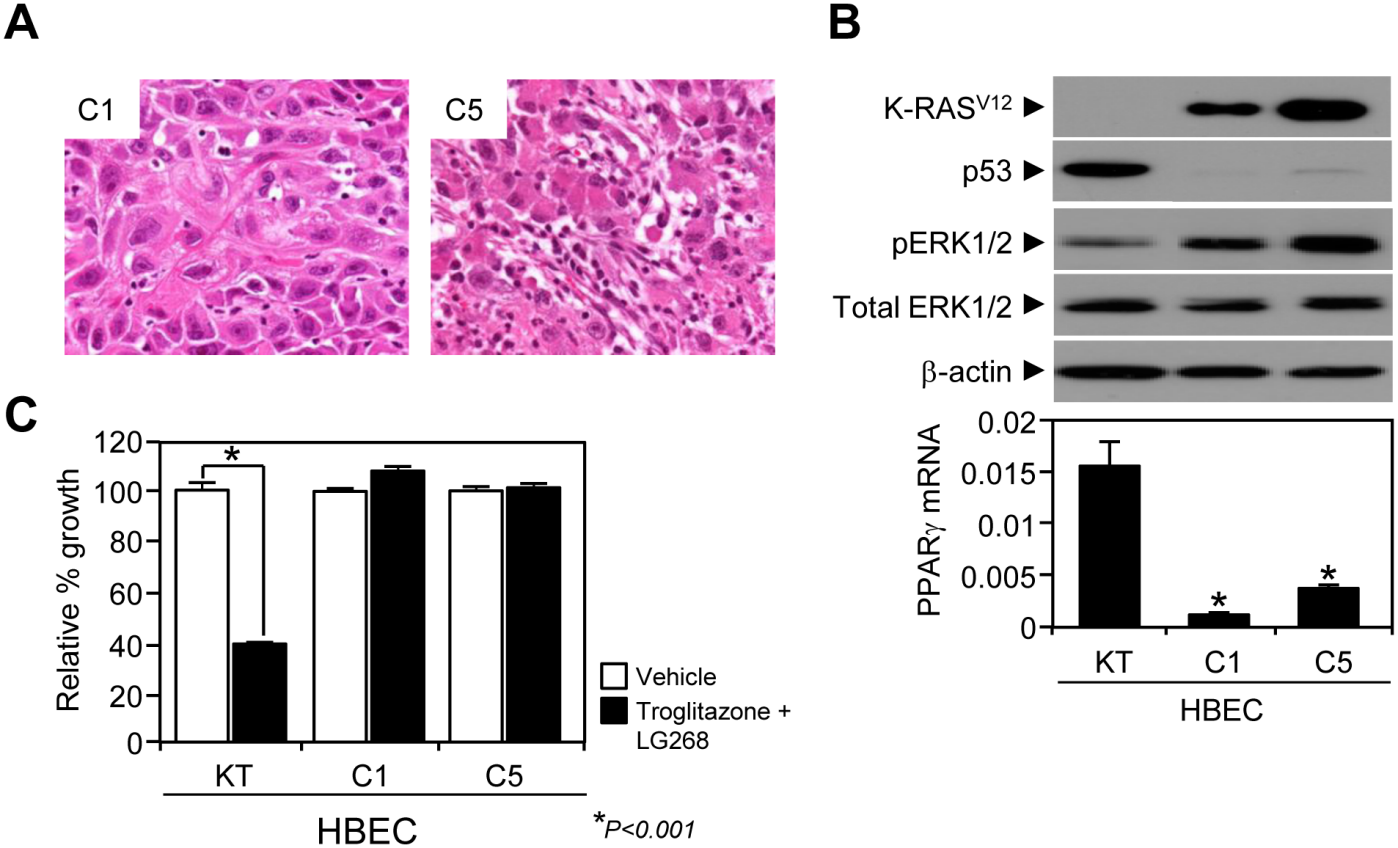
Characterization of tumorigenic HBEC clones. (A) Histologic analysis of tumorigenic clones; C1 tumor poorly differentiated carcinoma with large cells suggestive of squamous cell carcinoma (H&E, x40) (left) and C5 tumor poorly differentiated carcinoma with features of adenocarcinoma (H&E, x40) (right). (B) *In vitro* characterization of HBEC tumorigenic clones C1 and C5. Immunoblot assays were performed using antibodies against K-ras, p53, pERK, total ERK, and beta-actin in tumorigenic HBEC clones. Using QPCR assay, the mRNA expression of PPARγ was measured in tumorigenic clones (bottom in B). (C) Growth response of tumorigenic HBEC clones to the combined treatment of PPARγ (3 μM troglitazone) and RXR (100 nM LG268) ligands. Data represent the mean ± SD (n = 3). Asterisks show a statistically significant point as evaluated by *ANOVA*. * *P* < 0.001 compared to HBEC-KT control.

Since different levels of PPARγ expression reflected no difference in growth inhibition of non-tumorigenic HBECs when treated with troglitazone (Fig 3C), we wondered if the transformed HBECs show a different response to the PPARγ ligand treatment and distinct expression patterns of other NRs compared to the pre-cancerous HBECs. We first characterized the two tumorigenic clones, HBEC-C1 and -C5, at the cellular and tissue levels. Histological characterization of the xenograft tumors revealed, as shown recently by us, that HBEC-KT with p53 knockdown and K-ras^V12^ high expression manipulation could lead to clonal derivatives with different histologies- squamous cell carcinoma (SCC, HBEC-C1) and adenocarcinoma (ADK, HBEC-C5) [31](Fig 4A). Biochemical analysis confirmed that both oncogenic alterations, *K-ras* ^*V12*^ activity as well as loss of *p53* expression, were equally maintained in the tumorigenic HBEC-C1 and -C5 clones (Fig 4B). Surprisingly, both PPARγ and COX2 expressions were dramatically decreased in tumorigenic HBEC clones (Fig 4B and S3 Fig) and the tumorigenic clones were consistently resistant to PPARγ growth inhibition (Fig 4C). These data suggest the possibility that during premalignancy driven by *p53* and *K-ras* oncogenic changes that PPARγ and COX2 can be therapeutic targets but that with the development of full malignancy that the tumors bypass this control, which in some cases occurs by down regulation of PPARγ. Such findings would be consistent with reports that the use of non-steroidal anti-inflammatory drug (NSAID) can protect against the development of lung cancer in men while their use in fully developed lung cancers appears not to be therapeutic [40].

### NR expression in tumorigenic HBEC clones

**Fig 5:**
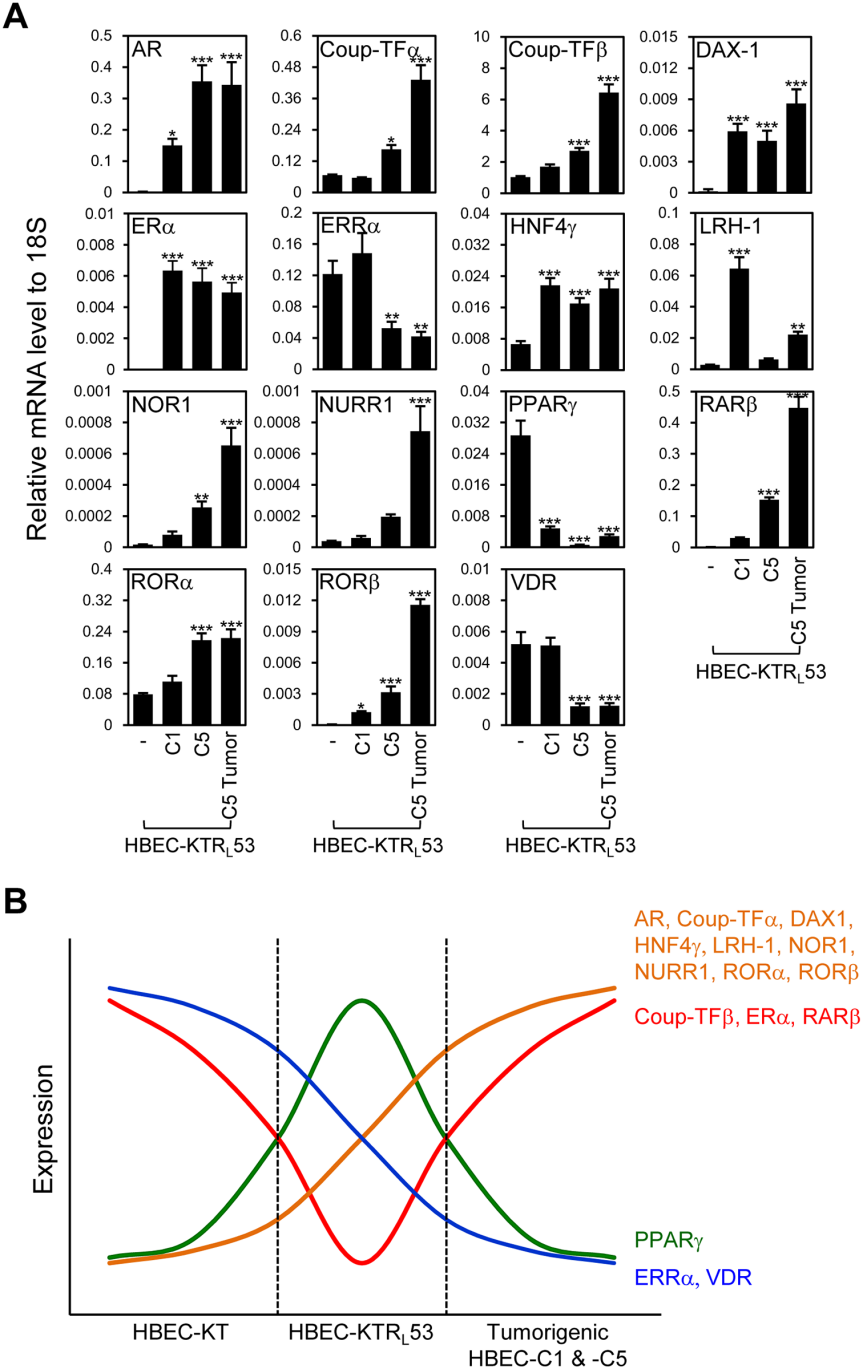
Expression profile of the NR superfamily in tumorigenic HBEC clones. The QPCR assay was performed for mRNA expression of the entire NR superfamily in non-tumorigenic HBEC-KTR_L_53 cells with *p53* and *K-ras* ^*V12*^ changes, two tumorigenic clones C1 and C5, and xenograft C5 tumor tissue. (A) Quantitative mRNA expression profiles of the NR subgroups with distinct expression pattern across the panel. Note that the rest of the NR profile was shown in S4 Fig. (B) Summary of NR expression from panel A and Fig 2 to show NR expression cascades correlated with tumorigenic progression. Data represent the mean ± SD (n = 3). Asterisks show statistically significant points as evaluated by *ANOVA*. *P < 0.05, **P < 0.01 and ***P < 0.001 compared to HBEC-KTR_L_53.

Since PPARγ expression was remarkably suppressed in the fully malignant HBECs, we wondered if any other NRs are differently expressed with the same tumorigenic progression. Thus, we completed the mRNA expression profile of the entire NR superfamily in the panel of non-tumorigenic HBEC-KTR_L_53 cells and the tumorigenic clonal derivatives including tumorigenic cell lines established from C1 and C5 tumors, and C5 tumor itself (Fig 5A and S4 Fig). Fifteen of the 50 NRs showed expression patterns potentially associated with the malignant progression of HBEC-KTR_L_53 cells into HBEC tumors (Fig 5A). Included in this group were AR, Coup-TFα, Coup-TFβ, dosage-sensitive sex reversal-adrenal hypoplasia congenital critical region on the X chromosome, gene 1 (DAX1), ERα, ERRα, HNF4γ, liver receptor homolog-1 (LRH1), NOR1, NURR1, PPARγ, RARβ, RORα, RORβ, and VDR (Fig 5A). Interestingly, nine out of the 15 NRs (AR, Coup-TFα, DAX1, HNF4γ, LRH1, NOR1, NURR1, RORα, and RORβ) showed continuously increasing expression pattern upon tumorigenic progression (Fig 5B). AR and DAX1 showed dramatically increased expression only in HBEC tumors, but not in the immortalized HBEC cell lines. ERRα and VDR showed decreased expression during tumorigenesis from non-oncogenic HBEC-KT, through HBEC-KTR_L_53 to HBEC tumors (Fig 5B). Coup-TFβ, ERα, and RARβ showed a biphasic expression pattern, where the initial expression of the NRs in HBEC-KT decreased in HBEC-KTR_L_53, but rebounded in HBEC tumors, which is opposite to the complete loss of PPARγ expression in HBEC tumors (Fig 5B). More interestingly, adenocarcinoma type HBEC-C5 cells and tumor, but not the squamous cell carcinoma HBEC-C1, showed increased expression of Coup-TFα and β, and decreased expression of ERRα and VDR, suggesting that these four NRs might be specifically involved in adenocarcinoma type-specific lung cancer pathogenesis.

### PPARγ sumoylation-dependent inhibition of tumorigenic HBEC growth and migration

**Fig 6:**
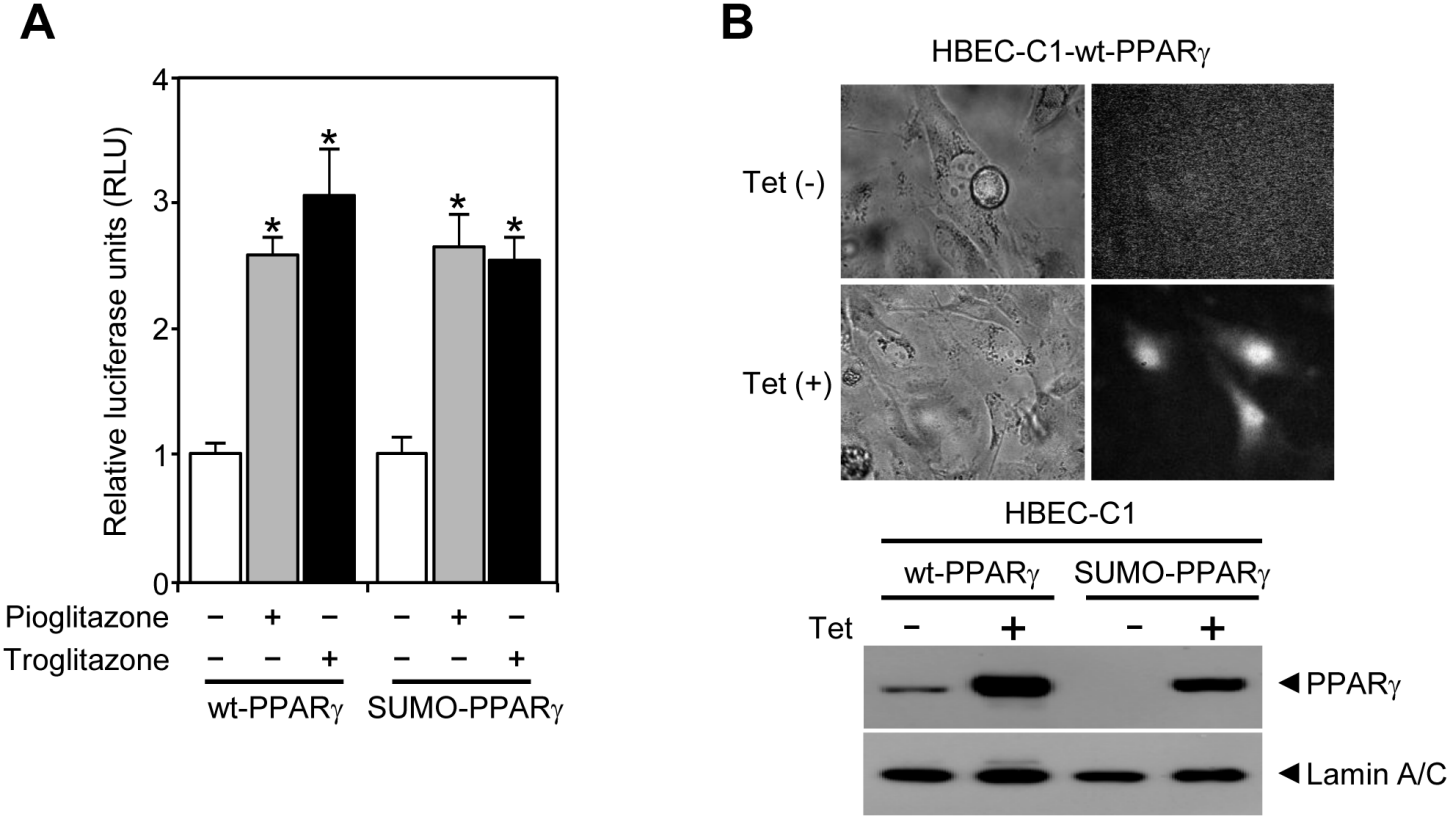
Characterization of wildtype and sumoylation mutant PPARγ. (A) Luciferase reporter assay of wildtype and sumoylation mutant PPARγ plasmids. RLU, relative luciferase unit. (B) Tetracycline-induced expression of PPARγ and EGFP protein in stably transfected HBEC-C1-wt-PPARγ clone. A microscopic view of tetracycline-induced EFGP expression (top in B). Immunoblot assays for the expression of lamin A/C and tetracycline-induced PPARγ (bottom in B). A bicistronic construct of PPARγ and EGFP was stably introduced into a tumorigenic HBEC clone to generate HBEC-C1-PPARγ cell lines as described in Materials and Methods. Data represent the mean ± SD (n = 3). Asterisks show statistically significant points as evaluated by *ANOVA*. *P < 0.001 compared to HBEC-KT control.

**Fig 7:**
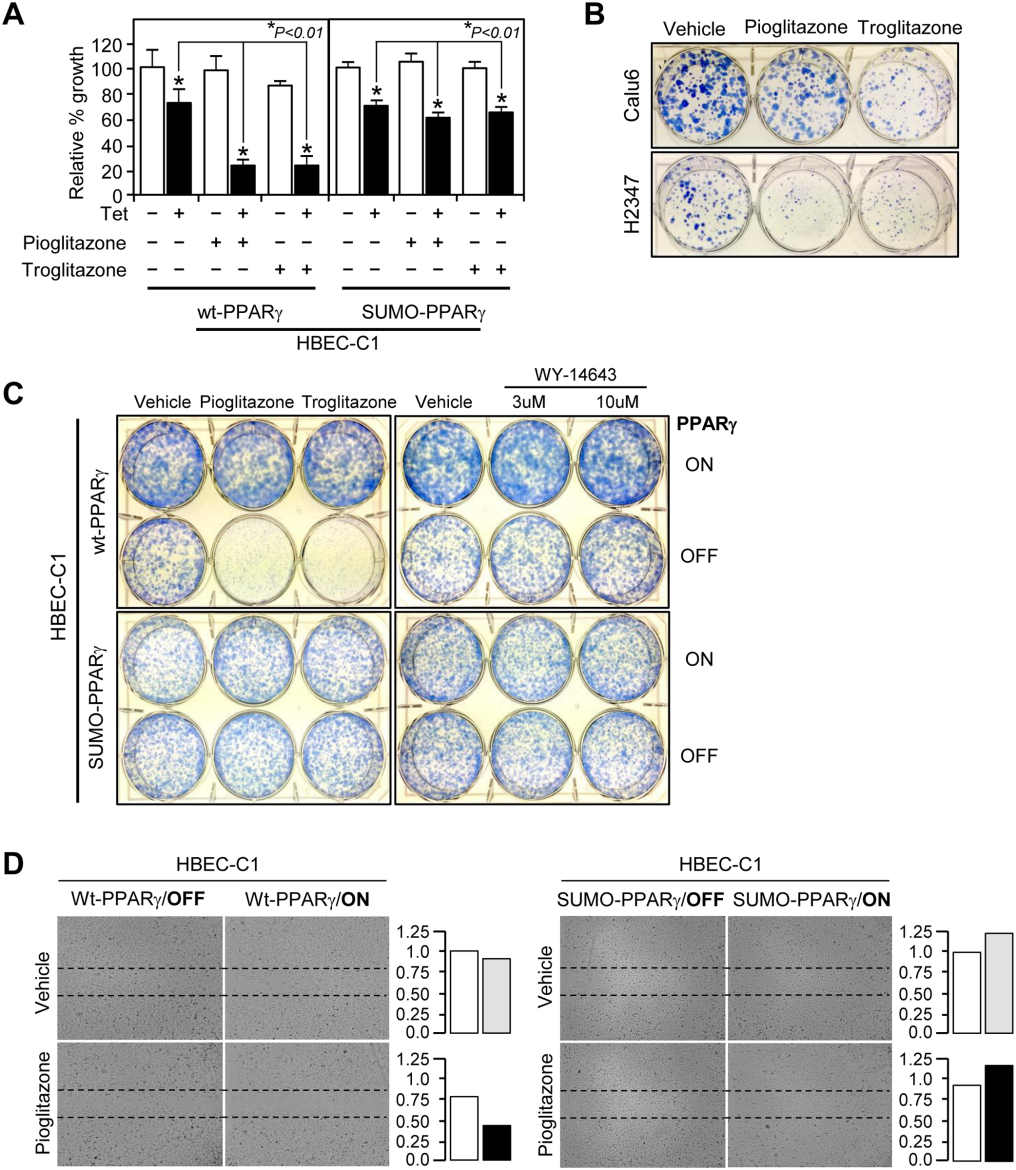
Functional evaluation of PPARγ in tumorigenic lung cancer cells. Growth response and cell migration of lung cancer were analyzed to treatment with PPARγ ligands. (A) Cell growth response. HBEC-C1-wt-PPARγ and HBEC-C1-SUMO-PPARγ cell lines were treated with 3 μM of pioglitazone or troglitazone for 3 days with or without tetracycline induction and followed by cell counting assay. Note that the result represents three independent sets of experiments. Liquid colony formation (B,C) and cell migration (D) assays were performed as described in Materials and Methods. Cells were treated with 3 μM of TZDs, or 3 and 10 μM of WY-14643 under the condition of PPARγ-ON or -OFF. Data represent the mean ± SD (n = 3). Asterisks show statistically significant points as evaluated by *ANOVA*. *P < 0.001 compared to HBEC-KT control.

**Fig 8:**
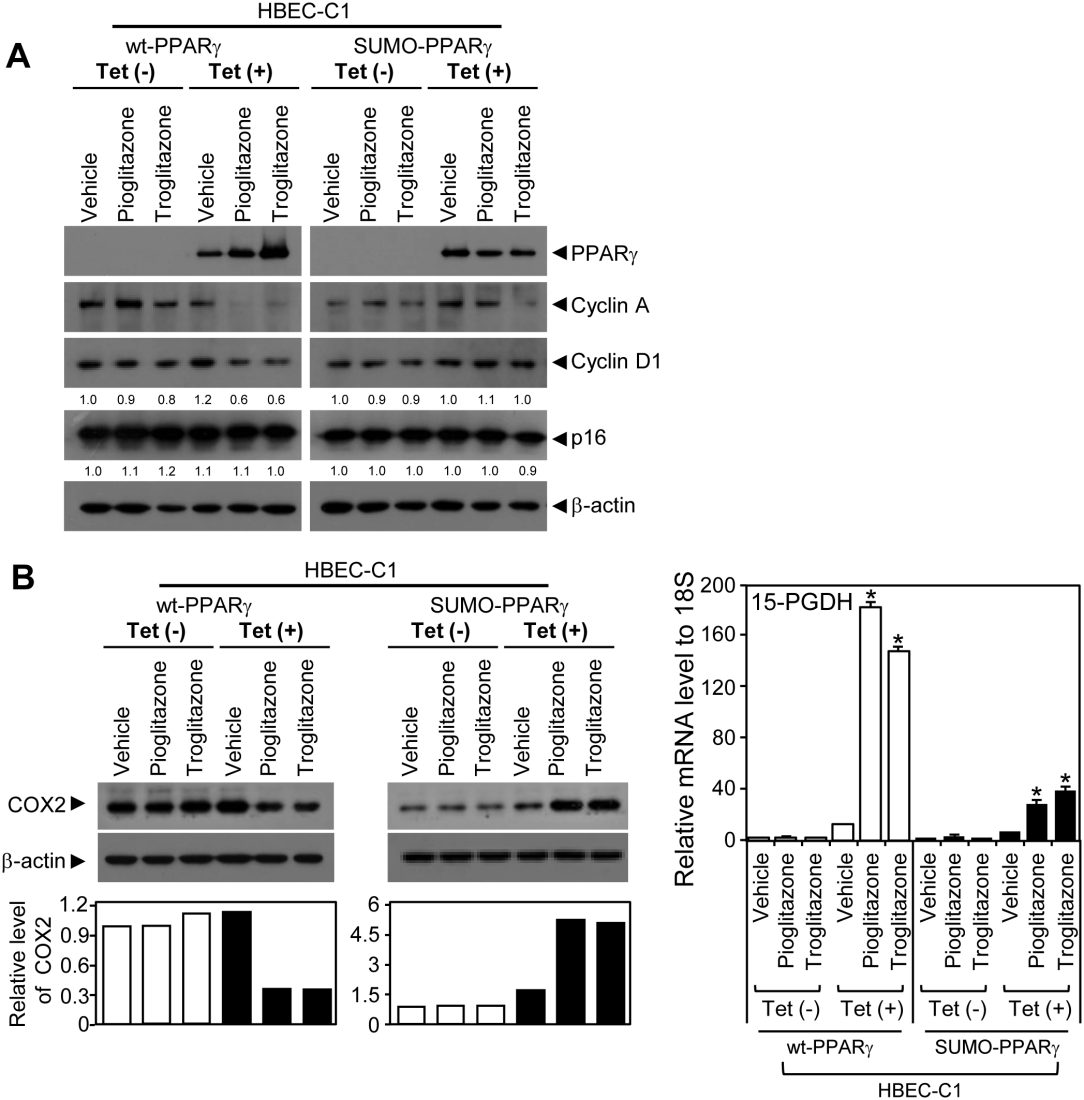
Expression of cellular and inflammatory factors in tumorigenic HBEC clones inducibly expressing PPARγ (A) Immunoblots using antibodies against PPARγ, cyclin A, cyclin D1, p16, or beta-actin were used to measure the corresponding protein expression in tumorigenic HBECs expressing wt-PPARγ or SUMO-PPARγ when treated with 3 μM of TZDs. (B) COX2 (left) and 15-PGDH (right) expression upon activation of wt-PPARγ or SUMO-PPARγ with 3 μM of TZD treatments. The expression of COX2 protein was measured by immunoblot assay in HBEC-C1 cell lines, HBEC-C1-wt-PPARγ and HBEC-C1-SUMO-PPARγ, treated with 3 μM of TZDs (e.g., pioglitazone or troglitazone) under tet-ON or -OFF condition. The relative level of COX2 expression was referenced by the corresponding beta-actin level and further normalized by the vehicle-treated sample in each group (left-bottom in B). 15-PGDH mRNA expression was measured using QPCR assay in the same sample sets (right panel in B). Data represent the mean ± SD (n = 3). Asterisks show statistically significant points as evaluated by *ANOVA*. *P < 0.001 compared to HBEC-KT control.

As ligand-mediated PPARγ sumoylation suppresses the expression of inducible nitric oxide synthase (iNOS), a well-known proinflammatory enzyme that generates nitric oxide, and the anti-inflammatory role of PPARγ is believed to contribute to its tumor suppressive function of that receptor [34], we tested whether PPARγ sumoylation is critical for the anti-tumorigenic function of that receptor in the HBEC progression series. To do this, we established tumorigenic stable cell lines (HBEC-C1) expressing the wild-type (wt-PPARγ) or sumoylation mutant (SUMO-PPARγ) form of PPARγ to study if gain-of-function of different forms of PPARγ (wt-PPARγ vs. SUMO-PPARγ) could reverse the growth resistance of the tumorigenic HBEC-C1 to ligand treatment. We identified that both PPARγ forms showed no difference in ligand-mediated trans-activation function for target gene expression using a luciferase assay (Fig 6A). HBEC-C1 cells tightly regulated the expression of wt-PPARγ or SUMO-PPARγ under the control of tetracycline-inducible operating promoter (Tet/ON) (Fig 6B). HBEC-C1 cells with induced expression of wt-PPARγ showed significant growth inhibition by more than 50% when treated with pioglitazone or troglitazone (TZDs) (Fig 7A). However, this growth inhibitory response was greatly diminished in HBEC-C1 cells with inducible expression of SUMO-PPARγ under the same treatment conditions as the corresponding HBEC-C1 cells expressing wt-PPARγ (Fig 7A and 7B). Similarly, liquid colony formation assay showed that PPARγ ligand treatments inhibited clonogenecity of HBECs expressing wt-PPARγ and other lung cancer cell lines such as calu6 and H2347 expressing endogenouse PPARγ, but not of the one with SUMO-PPARγ (Fig 7B and 7C). However, treatment of PPARα ligand WY-14643 showed no colonogenic effect in both wt-PPARγ and SUMO-PPARγ expressing HBECs (Fig 7C). This suggests that inhibitory effect of TZDs is specifically dependent on PPARγ sumoylation. PPARγ activation also inhibited cell migration in a sumoylation-dependent manner (Fig 7D). Consistent with this, the expression of both cyclin A and cyclin D1 decreased in HBEC-C1 cells expressing wt-PPARγ, but was not changed in HBEC-C1 cells expressing SUMO-PPARγ, when treated with TZDs (Fig 8A and S5A Fig). Note that the expression of cyclin-dependent kinase inhibitors, p16 and p21, were not significantly changed in the same treatment conditions (Fig 8A and S5A Fig). In addition, we investigated several inflammatory signaling pathways involved in nitric oxide production, prostaglandin biochemistry, and TNFα signal transduction. Intriguingly, we found that TZD activation of wt-PPARγ decreased the proinflammatory COX2 expression by three-fold, whereas SUMO-PPARγ activation, notably, increased the expression of COX2 protein by six-fold in HBEC-C1 cells (Fig 8B). Further, the expression of 15-hydroxyprostaglandin dehydrogenase (HPGD), a prostaglandin-metabolizing enzyme, was dramatically induced by eighteen-fold when the wt-PPARγ was activated by TZDs. However, HBEC-C1-SUMO-PPARγ cells induced the HPGD expression significantly less (five- to seven-fold) when treated with TZDs. PPARγ activation also significantly suppressed TNFα expression, but not other NFκB signaling factors, in a sumoylation-dependent manner (S5B Fig). Note that iNOS was not expressed in tumorigenic HBEC clones (S5C Fig). Taken together, this suggests that ligand-induced PPARγ sumoylation is specifically involved in the suppression of inflammatory COX2 and TNFα signaling pathways, but not the iNOS pathway, in lung cancer pathogenesis.

## Discussion

We have previously demonstrated that the NR superfamily provides a set of prognostic biomarkers as well as potential theragnostic (e.g. diagnostic and therapeutic) targets for lung cancer [7, 8]. These findings prompted us to further investigate the pathogenic involvement of the NR superfamily in lung cancer progression. In the present study, to demonstrate that NRs are involved in oncogene-induced lung cancer pathogenesis, we have performed several independent molecular and biochemical approaches: 1) to investigate and analyze the mRNA expression of the entire NR superfamily in the oncogenesis model; 2) to study the molecular function of a particular nuclear receptor PPARγ, as a proof-of-principle study; and 3) to interrogate the function of biochemically modified PPARγ in that sumoylation of the receptor is critical for inhibiting the expression of proinflammatory COX2 as well as TNFα, cell cycle proteins, and cell migration, thereby contributing to tumor suppression in lung cancer. To that end, we first utilized an isogenic panel of human bronchial epithelial cells that consisted of both precancerous and tumorigenic HBECs. It is worthwhile to note that HBECs in this study were derived from a smoker, suggesting that this panel could represent both a smoking and genetic model for studying human lung cancer pathogenesis. Thus, one of the key advances in this work was to utilize a complete set of human lung cancer pathogenesis models, where isogenic clones represented a stepwise progression of normal HBECs into precancerous, and finally malignant HBECs. In this study, we further established tumorigenic HBECs with inducible expression of PPARγ for studying biochemical modification-associated anti-tumorigenic function of that receptor.

Although diverse animal models, including a genetic and chemical carcinogen models mimicking smoking, have been extensively utilized for genetic or epigenetic studies of lung cancer, understanding oncogenesis in human lungs remains as a concern [41–43]. In this regard, we believe that this panel could represent the first human pathogenesis model to study genetic as well as environmental causes (e.g. smoking) of lung cancer. Notably, we found that the two tumorigenic clones C1 and C5 represented SCC and ADK, respectively, the major subtypes of non-small cell lung carcinoma. This suggests that the two oncogenic mutations, *K-ras* ^*V12*^ and *p53* knockdown, are sufficient to induce cancer progression into lung adenocarcinoma, while smoking appears to be necessary for the development of SCC. This is consistent with previous reports that transgenic mouse models, *K-ras* alone or together with *p53* knockout, mainly developed ADK. Therefore, it will be intriguing to treat the mouse genetic model with chemical carcinogens mimicking smoking to see if SCC develops.

Secondly, from the comparative analysis of the mRNA expression of the entire human NR superfamily in the HBEC model, subsets of NRs were identified as showing distinct expression patterns upon oncogenesis (Fig 5A). Closer investigation of the analysis revealed that the expression pattern of the 15 NRs was remarkably well-fitted into the two clusters that we found previously by analyzing the NR expression pattern in 55 lung cell lines. The two clusters represented NRs over- or under-expressed in lung cancer cell lines when normalized by normal HBECs [7]. Furthermore, four out of the 15 NRs—ERRα and VDR with reduced expression and Coup-TFα and β with increased expression- showed an adenocarcinoma specific expression pattern. ERRα is a notable example since it plays an important role, along with protein ligand peroxisome proliferator-activated receptor γ coactivator-1α (PGC1α), in controlling mitochondria biogenesis and function [44]. Therefore, one might think that energy homeostasis could be dysregulated in ADK compared to SCC, and this provides a rationale for further mechanistic studies to metabolically differentiate ADK from SCC, particularly linked to smoking. In addition to finding adenocarcinoma specific NRs, the finding that the 15 NRs were similarly dysregulated in lung cancer cell lines suggests that homeostatic balance of the transcriptional network of NRs plays an important role in preventing lung cancer pathogenesis. Together with our previous reports of NR involvement in lung cancer prognosis, diagnosis, and therapeutic potential, this study additionally revealed that subsets of NRs might be involved in oncogene-induced lung cancer incidence, indicating that NRs are significant in the onset as well as maintenance of lung cancer. Given the idea that NRs are druggable targets for various diseases, NRs can be managed prophylactically to prevent lung cancer incidence as well as be developed into therapeutic targets. Indeed, several clinical studies targeting PPARγ have been executed recently to treat advanced lung cancer patients since many preclinical lung cancer studies have proposed the anti-inflammatory function and therapeutic potential of PPARγ for treating lung cancer [7, 34, 35, 38, 39]. However, some reports previously argued that PPARγ activation is non-tumor suppressive in prostate cancer, and even promotes tumorigenesis in breast cancer [28–30]. In this regard, a last notable finding from this work was that ligand-mediated sumoylation is critical for anti-tumorigenic function of PPARγ in lung cancer. Consistent with the previous report of PPARγ suppression of iNOS expression, we similarly observed that ligand-mediated, sumoylated PPARγ decreased the expression of COX2 and TNFα, and thus suppressed tumor cell migration and growth. However, sumoylation mutant PPARγ showed no prominent cellular growth response as well as migration when treated with TZDs. This suggests that TZDs would be applicable to the tumors in which the PPARγ expression with no mutation of sumoylation sites should be assessed for clinical application of TZDs to the cancer patients. This screening process would improve the drug response for those particular subsets of patients and even be able to treat lung cancer patients with type II diabetes with a single PPARγ ligand. Furthermore, this provides an insight into the effects of biochemical modification of NRs for understanding different physiological and pathological responses upon ligand treatment [45]. For example, along with sumoylation, PPARγ phosphorylation could play an important role in lung cancer pathogenesis as fibroblast growth factor (FGF) activated- or cyclin-dependent kinase 5 (CDK5) phosphorylation of PPARγ revealed pathologically or physiologically altered PPARγ signaling [46–49].

Collectively, our studies support the idea that tumorigenesis could be attributed to different biochemical modifications of NRs upon ligand binding, which consequently become selective nuclear receptor modulators such as selective PPARgamma modulator (SPARM) or SERM [19, 23, 24, 49]. By profiling the 48 NRs, investigating ligand mediated biochemical modifications, and preclinically validating the pathogenic function of the selected NRs in an oncogenesis model, our work provides a new strategy to develop chemopreventive as well as therapeutic approaches for cancer clinics in the future.

## Supporting Information

S1 Fig QPCR assay for p53 mRNA expression in immortalized HBECs. The p53 knockdown was confirmed in HBECs stably transfected with short hairpin plasmid for p53.
(TIF)

S2 Fig Expression profile of the NR superfamily in immortalized HBEC panel. The QPCR assay was performed to measure mRNA expression of the NR superfamily in the immortalized HBEC panel. Thirty-one NRs showed no expression or no change in the expression upon oncogenic alterations. (A) The sixteen NRs with no expression include AR, CAR, DAX1, ERRβ, ERRγ, FXR, HNF4α, LRH1, PNR, PPARγ2, PR, PXR, RXRγ, SF1, SHP, and TLX. (B) The fifteen NRs with no change include Coup-TFγ, GR, LXRα, LXRβ, MR, RARα, RARγ, Rev-erbβ, RORγ, RXRα, RXRβ, TR2, TR4, TRα and VDR. The x-axis represents cell names and the y-axis represents relative mRNA expression of the corresponding NR.
(TIF)

S3 Fig Expression of PPARγ and COX2 in the panel of precancerous and tumorigenic HBEC cells. The mRNA expression of PPARγ and COX was measured using QPCR assay in immortalized as well as tumorigenic HBEC clones, C1 and C5. The x-axis represents HBEC cell names and the y-axis represents relative mRNA expression of the genes of interest.
(TIF)

S4 Fig Expression profile of the NR superfamily in tumorigenic HBEC clones. Using QPCR assay, the mRNA expression profile of the NR superfamily was surveyed in tumorigenic HBEC clones. Thirty-five NRs showed no distinct expression patterns: fifteen NRs were in the low or no expression group (A), and twenty NRs were expressed but expression did not change upon oncogenesis (B). The x-axis represents cell names and the y-axis represents relative mRNA expression of the corresponding NRs.
(TIF)

S5 Fig mRNA expression of cellular factors involved in cell cycle and inflammation upon PPARγ sumoylation. The QPCR assay was performed to measure the mRNA expression of cellular factors involved in cell cycle progression (A) and inflammatory responses (B, C). HBEC-C1-wt-PPARγ and HBEC-C1-SUMO-PPARγ cell lines were treated with 3 μM of pioglitazone or troglitazone under tetracycline ON or OFF condition. Note that iNOS expression was not detectable in HBEC-C1 cells (C). The x-axis shows treatment conditions and the y-axis represents relative mRNA expression of the genes of interest. Data represent the mean ± SD (n = 3). Asterisks show statistically significant points as evaluated by *ANOVA*. *P < 0.05, **P < 0.01 and ***P < 0.001 compared to vehicle control.
(TIF)
