# Supplementary figures and images for: Nuclear Receptor Expression and Function in Human Lung Cancer Pathogenesis
Source: PLoS One. 2015 Aug 5;10(8):e0134842. doi: 10.1371/journal.pone.0134842 (PMC4526668; doi:10.1371/journal.pone.0134842)

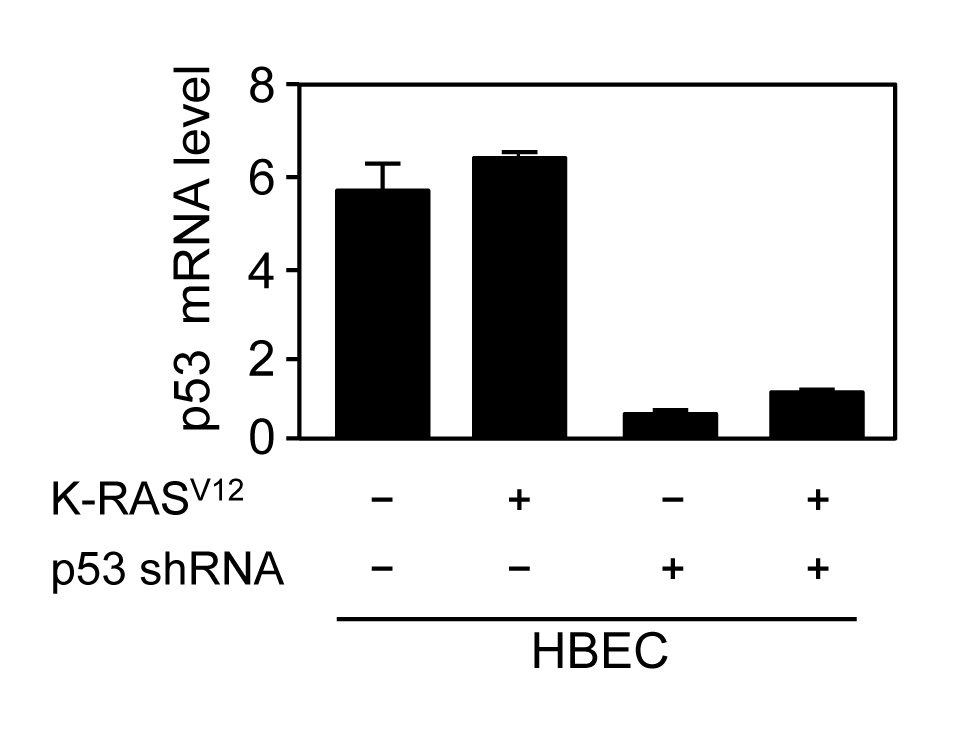

Supplement: S1 Fig — The p53 knockdown was confirmed in HBECs stably transfected with short hairpin plasmid for p53. (TIF) [file pone.0134842.s001.tif]

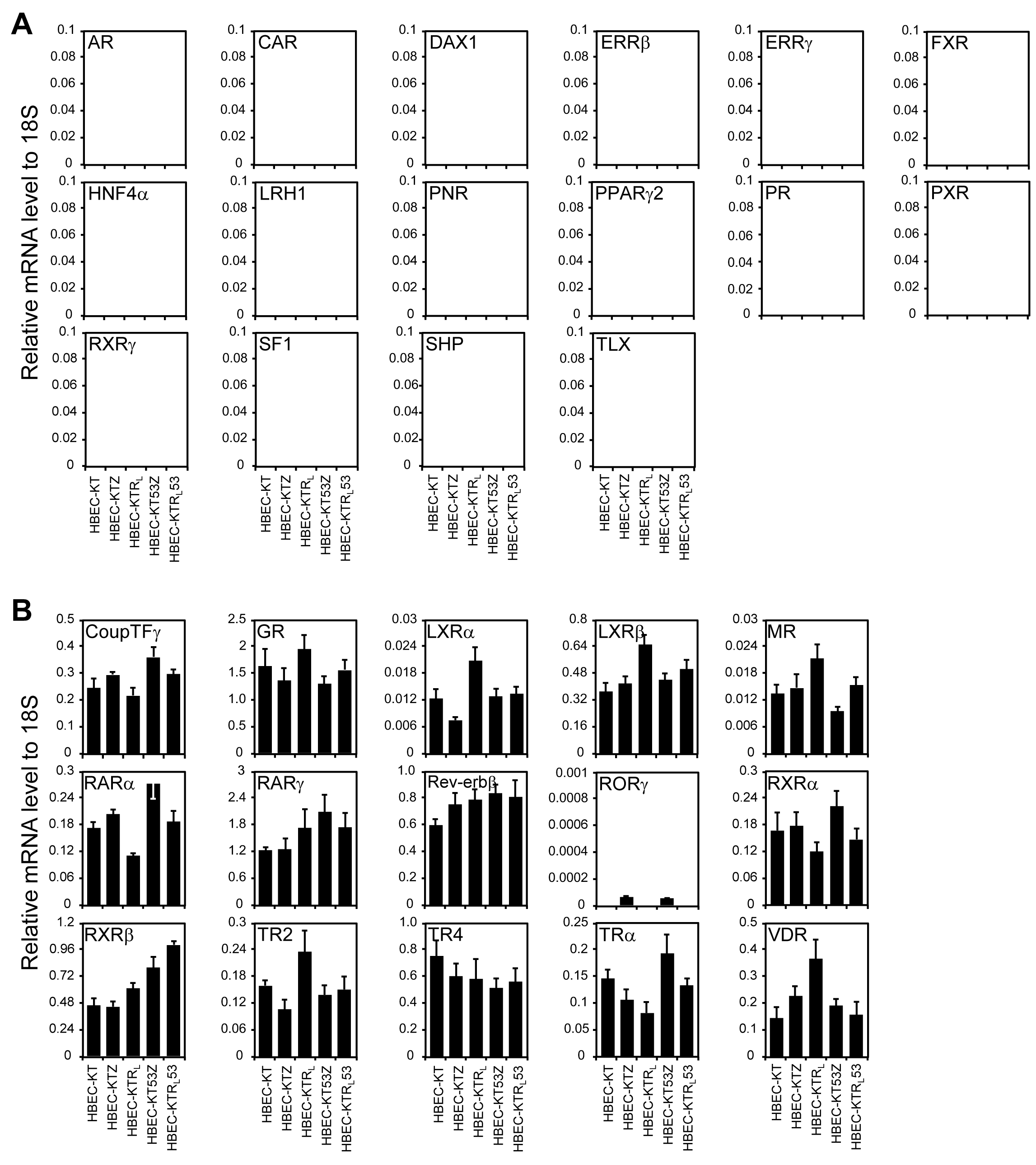

Supplement: S2 Fig — The QPCR assay was performed to measure mRNA expression of the NR superfamily in the immortalized HBEC panel. Thirty-one NRs showed no expression or no change in the expression upon oncogenic alterations. (A) The sixteen NRs with no expression include AR, CAR, DAX1, ERRβ, ERRγ, FXR, HNF4α, LRH1, PNR, PPARγ2, PR, PXR, RXRγ, SF1, SHP, and TLX. (B) The fifteen NRs with no change include Coup-TFγ, GR, LXRα, LXRβ, MR, RARα, RARγ, Rev-erbβ, RORγ, RXRα, RXRβ, TR2, TR4, TRα and VDR. The x-axis represents cell names and the y-axis represents relative mRNA expression of the corresponding NR. (TIF) [file pone.0134842.s002.tif]

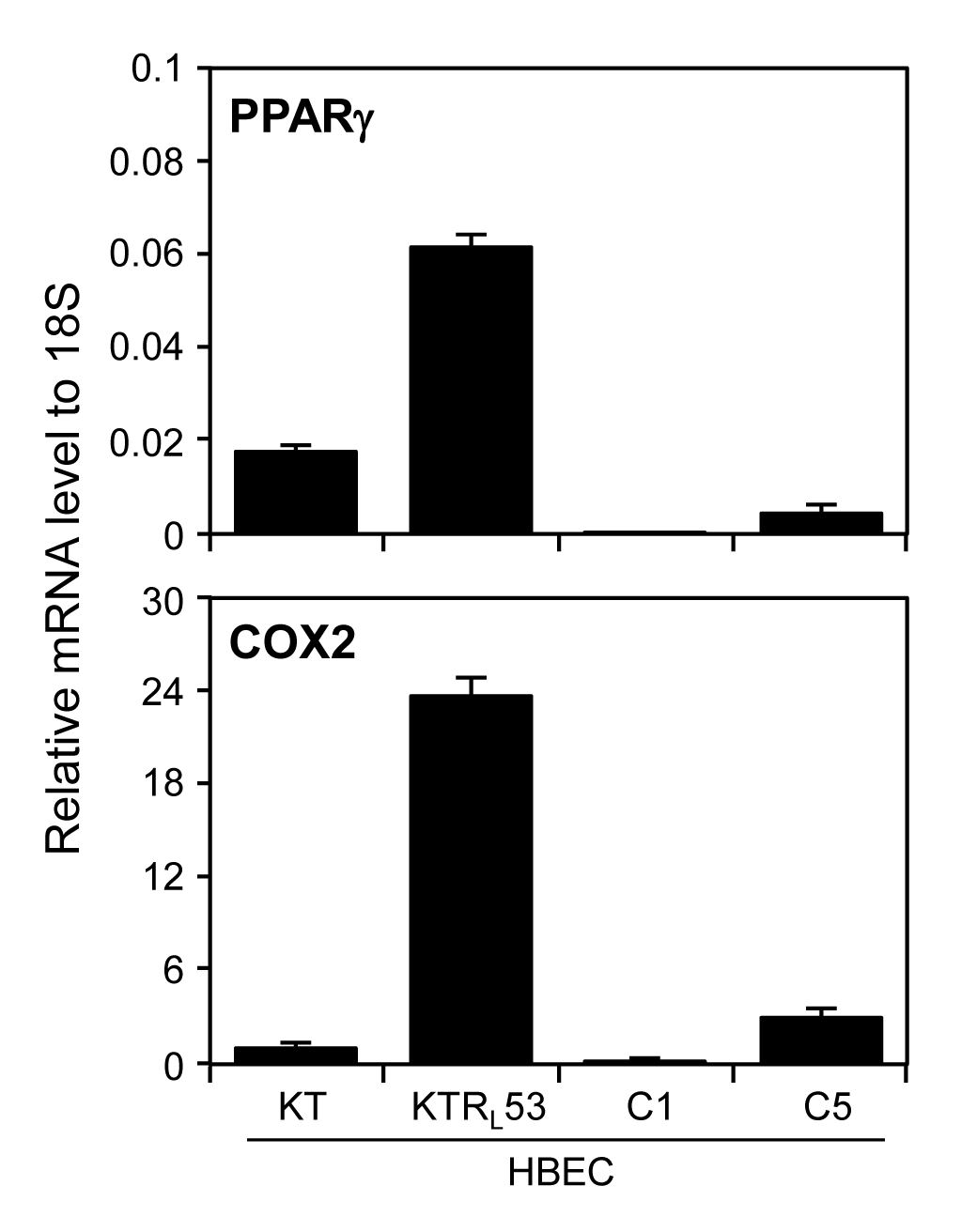

Supplement: S3 Fig — The mRNA expression of PPARγ and COX was measured using QPCR assay in immortalized as well as tumorigenic HBEC clones, C1 and C5. The x-axis represents HBEC cell names and the y-axis represents relative mRNA expression of the genes of interest. (TIF) [file pone.0134842.s003.tif]

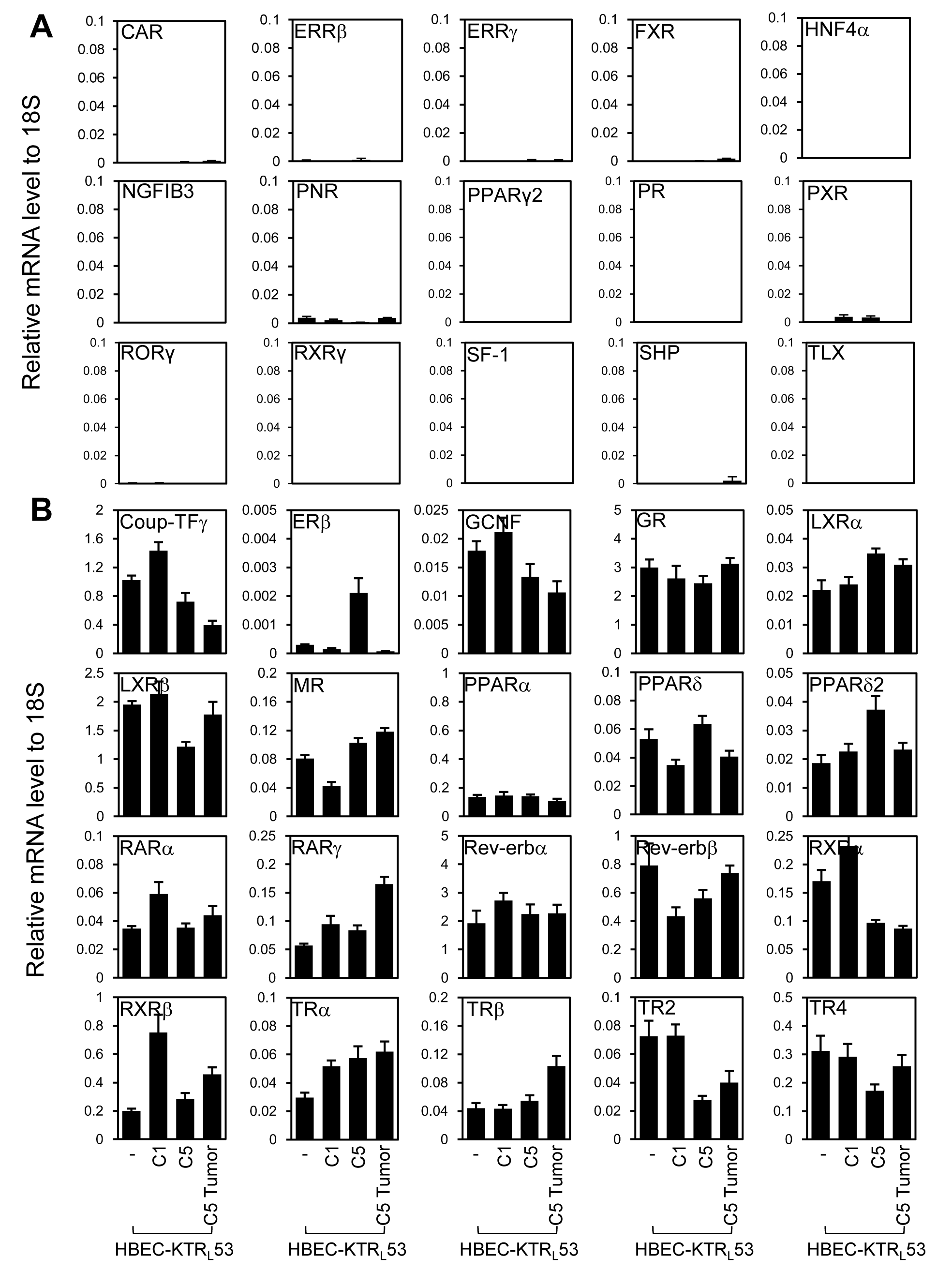

Supplement: S4 Fig — Using QPCR assay, the mRNA expression profile of the NR superfamily was surveyed in tumorigenic HBEC clones. Thirty-five NRs showed no distinct expression patterns: fifteen NRs were in the low or no expression group (A), and twenty NRs were expressed but expression did not change upon oncogenesis (B). The x-axis represents cell names and the y-axis represents relative mRNA expression of the corresponding NRs. (TIF) [file pone.0134842.s004.tif]

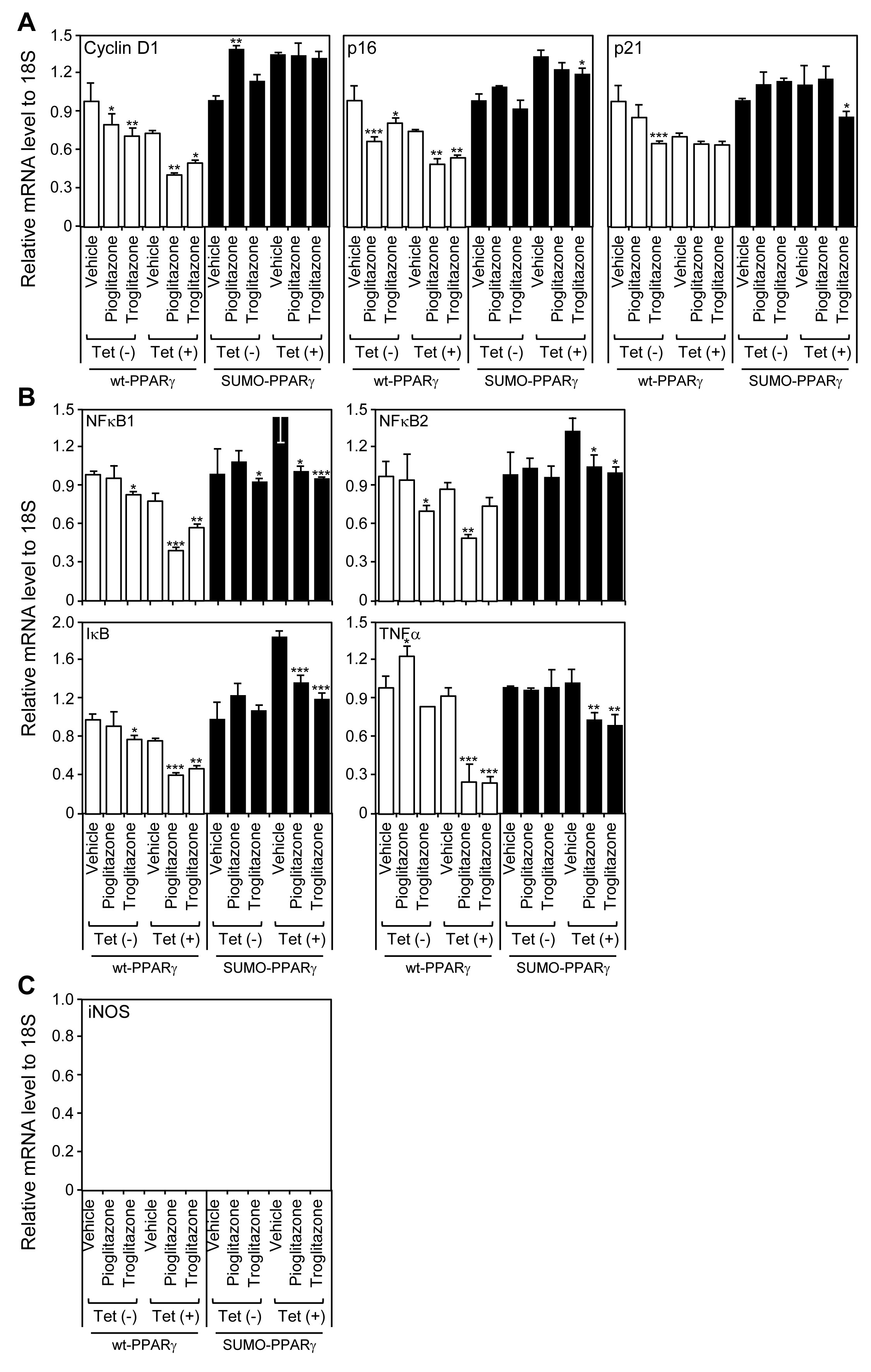

Supplement: S5 Fig — The QPCR assay was performed to measure the mRNA expression of cellular factors involved in cell cycle progression (A) and inflammatory responses (B, C). HBEC-C1-wt-PPARγ and HBEC-C1-SUMO-PPARγ cell lines were treated with 3 μM of pioglitazone or troglitazone under tetracycline ON or OFF condition. Note that iNOS expression was not detectable in HBEC-C1 cells (C). The x-axis shows treatment conditions and the y-axis represents relative mRNA expression of the genes of interest. Data represent the mean ± SD (n = 3). Asterisks show statistically significant points as evaluated by ANOVA. *P < 0.05, **P < 0.01 and ***P < 0.001 compared to vehicle control. (TIF) [file pone.0134842.s005.tif]
